# Hospital and Institutional News

**Published:** 1915-04-03

**Authors:** 


### HOSPITAL AND INSTITUTIONAL NEWS.

### THE WAR OFFICE AND THE POOR LAW.

The workhouse of the City of London Union at Homerton has been taken over by the War Office, and forty convalescent wounded soldiers were transferred to it last week from the London hospitals. The Fulham workhouse and infirmary have also been cleared of their inmates, and will probably receive wounded in the course of the next few days. The infirmaries and workhouses of the Mile End, Lewisham, and Hampstead Guardians, and the infirmary of the Bethnal Green Guardians have also been acquired by the military authorities, and steps are now being taken to transfer their present inmates. That so many Poor-Law institutions can thus be freed for military purposes is a striking indication of the economy which could be effected under normal circumstances by a combination of the London Unions. All the inmates displaced are being accommodated in the institutions of adjoining Unions. All the institutions thus taken over are to be administered as military hospitals, and, with a few exceptions, the staff is to be transferred with the institution. Care has been taken to safeguard the interests of the transferred officers. Administrative details have not yet been fully settled, and developments will be watched with a great deal of interest by Poor-Law officers. At present there are difficulties in the way of the complete severance of the institutions from the Guardians, and it is probable that for the present the latter will still retain control over the supply of food, drugs, and necessaries. Dual control, quite apart from sentimental objections to the connection of Poor-Law Guardians with hospitals intended solely for the use of sick and wounded soldiers, has many drawbacks, and the War Office will in the end probably assume the entire responsibility. One of the greatest difficulties will be the provision of the nursing staff necessary, and this will be the more easily surmounted if all connection with the Poor Law is severed.

### A SURGEON'S EXPERIENCES AS A PRISONER IN GERMANY.

All who want a straightforward, unvarnished account of the kind of treatment likely to be accorded to surgeons and other members of English Red Cross units on being captured by the Germans, could not do better than read Mr. L. J. Austin's "Experiences as a German Prisoner," now published by Mr. Melrose. There is nothing sensational in this little volume, but the story of his arrest with Dr. Elliott, and their transfer to prison quarters at Cologne, Torgau, Magdeburg, and other cities, leaves a very sharp impression of discomfort, annoyance, helplessness in the face of apparently capricious orders, and monotony of life. The descriptions should make the most insensitive person realise that imprisonment is a severe punishment under any circumstances, and when the captor and captive belong to nations engaged in war petty persecution is always liable to occur. One's wits may get sharpened in the process, as is shown by Mr. Austin's account of how the request of the commandant at Torgau to the English prisoners for subscriptions to the German Red Cross was ultimately agreed to in the hope, justified by events, that the Germans would take the trouble, for the sake of the cash, to send the cheques to England, and thereby provide a means of communicating with the families of those who signed them. The cheques were drawn on an Amsterdam bank, with requests to notify the writers' families written across them. The writer wisely refrains from going outside his subject, and touches on nothing of which his experience is not at first hand. By this self-denial he shows very effectually the isolation, petty interests, and ignorance of the world's affairs in which the punishment of imprisonment consists. To have had the experience is indeed a strange one for a Red Cross surgeon, and Mr. Austin, if he has seen none of the surgery of war, has made good use of his other opportunities during the four months of his detention. The book's price is 2s.

### NAVAL SURGEONS FOR THE ARMY.

The shortage of doctors with the Army and at home which has recently received official admission has produced a crop of suggestions for the better distribution of existing medical resources. All are worth attention, and perhaps not least the latest, put forward in *Truth*, to transfer to the Army a proportion of the naval surgeons "who are simply useless and idle." The suggestion is supported on the grounds that there are three surgeons in every ship, from the cruiser class upwards, at present with nothing to do; that the prospect of a general naval action is remote; that if it occurred there is not likely to be any obstacle to transferring wounded to a hospital ship or base within a few hours of action; that ship conditions practically limit a medical man to the practice of first-aid which an efficient sick-berth staff can perform under the superintendence of a single doctor; and, lastly, the grim but undoubted fact that the naval engagements of the present war have shown that it is a case of sink or be sunk, kill or be killed, and that while the loss of life has been heavy the proportion of wounded to killed has been extremely low. The contention, therefore, is that the whole of the temporary surgeons should be transferred from the Navy to the Army, and possibly some others also. That modern naval warfare has superseded the need for a large complement of naval surgeons would have had an air of paradox about it before the war; but is not the lesson of events that the Navy can largely dispense with their services, while the Army is seen to be more and more in need of medical men?

### CLAIM OF £15,000 AGAINST ST. GEORGE'S HOSPITAL.

The President and governors of St. George's Hospital were defendants in an action brought against them last week by Messrs. Wallrock and Co., estate agents, for £15,000 commission for services rendered in connection with the proposed sale of the hospital site to Mr. Mallaby-Deeley, M.P. The plaintiffs desired that the case should be postponed until the autumn on the ground that Mr. Wallrock had gone to Mexico on business and would not be back until July. Their case was that Mr. Wallrock introduced Mr. Mallaby-Deeley as a prospective purchaser of the hospital site, and that the sale failed to take place through reasons quite unconnected with the plaintiff, who contended that, nevertheless, he was entitled to the commission. The defendants opposed the application, and asked that the action should be disposed of as soon as possible, since it was prejudicial to the hospital authorities to have a claim for so large a sum hanging over their heads for an unreasonable length of time. The defence included a number of points of law and fact, among the latter of which was a question as to whether Mr. West, one of the then managers, had authority to make the contract sued upon. Mr. Justice Darling, in saying that the action would be in the paper on April 15, suggested that possibly the questions of law might be dealt with in the plaintiff's absence, and if decided against them there would be an end of the matter. The plaintiff was directed to pay the costs of the application.

### STAFF AND WAR SALARIES AT LEICESTER.

The governors of the Leicester Royal Infirmary are remarkable for having realised that hospital anniversary meetings can be made of lively public interest. At the recent anniversary meeting, presided over by Mr. H. Simpson Gee, the chairman of the board, the problems created by the war were presented in a manner of more than local appeal. In moving the adoption of the report, Mr. Gee emphasised the difficulties under which the infirmary had worked during the year. In the early months the reconstruction of the children's hospital had handicapped the institution, while in August war was declared, which had upset every department. The majority of the honorary medical and surgical staff, and many of the resident staff, left for service with the Forces, and this put the administration in difficulties. Owing to the shortage of medical service extra work had been occasioned to the nursing staff, already attenuated by war demands, and the governors were asked to acknowledge these special services by approving of an expenditure of £500 for increased salaries to the nurses. The board had placed the out-patients' department at the disposal of wounded soldiers, and Mr. Bond had patriotically undertaken the operative treatment of recruits rejected from the Services for physical defects, 120 of whom had already been dealt with.

### THE NEW CHAIRMAN'S POLICY.

Mr. Gee remarked at the same meeting that he would like to further the policy of increasing the endowment fund to provide for future needs and emergencies. The hospital now accommodated 300 patients, and all the departments were equal to the work required of them, so that they had a well-balanced hospital. Their expenses were heavy, and it would add greatly to the value of the £200,000 spent in buildings if they could create an endowment sufficient to pay the cost of management, etc., so that workpeople's contributions could be returned to them in maintenance and medical attendance clear of administrative expenses. The cloud on the horizon was the increased cost of all purchasable commodities and the knowledge that they were now paying for medical services which were formerly rendered gratuitously. Leicester was reputed to be making money—let some of it be given in extra help at this difficult time to Leicester's greatest charity. In referring to the value of the new children's hospital, Mr. Bond mentioned that, whereas in the last twelve months of the old building they had to close the wards eight times by reason of infection, since the new hospital had been opened—practically twelve months—there had not been a single outbreak, while the improved health of nurses and children had been noticeable. Referring to the great shortage of medical men, he urged the great field there was for medical women.

### NERVE HOSPITALS AND DRUGS.

As far as its accommodation allows, the Hospital for Epilepsy and Paralysis in Maida Vale has placed beds at the disposal of the military authorities, and some specially selected cases have been sent direct from Flanders or transferred from the military hospitals in this country. Mr. Charles Drummond, at the annual meeting last week, gave some figures as to the cost of drugs which will one day have historic interest, however much anxiety rather than interest is aroused concerning them at the present time. The hospital, he pointed out, was called upon to pay at present £4 12s. 6d. per oz., as compared with 17s. 3d. per oz. before the war. In the same way tabloids of aspirin had risen in price from 2s. to 12s. per thousand; while potassium bromide, "required by the hundredweight," since the war had been costing the hospital 7s. per lb., although a year or two ago it had been possible to purchase it at 9d. How far in the case of most commodities war prices are not being used to reimburse the manufacturers for war taxation rather than for any immediate rise in the cost of production caused by the outbreak of hostilities is a question very generally asked. But whatever be the true answer the prices have to be paid, and the hospitals must use the new proofs of their usefulness afforded by the war as a means for raising the necessary money.

### £150,000 FOR TWO HOSPITALS.

Through the support given by the late Sir Alfred Jones to the study of tropical medicine, the name of the Elder Dempster line of steamships has been already associated with hospitals. Another remarkable instance of the work for voluntary charity undertaken by the heads of this firm is seen in the will of the late Mr. Alexander Elder, founder of the line, who has bequeathed £100,000 upon trust to found, equip, and endow a hospital for the poor of Govan at or near the Elder Park, Govan, to be known as the "David Elder Infirmary," in memory of his father. He has also bequeathed £50,000 to the Western Infirmary, Glasgow, for a new wing to be called the "Alexander Elder Wing"; £3,000 to the Southport Infirmary; £2,000 each to the Southport Convalescent Hospital and Sea Bathing Infirmary and the Royal Southern Hospital, Liverpool; £1,000 to the Liverpool Maternity Hospital. The residue of his property he left for such charitable objects in the cities of Liverpool and Glasgow, or either of them, existing at his death as his executors may think fit, or for the founding of new charities in those cities. He expressed the wish that preference should be given to those for the benefit of seamen or engineers, or for poor lads to be trained for the sea, or the endowment of beds in hospitals or infirmaries; and if more than £50,000 is given to or expended upon any one object, he requested that his name might be associated with it.

### THE NEW PRESIDENT OF THE CORONERS' SOCIETY.

We heartily congratulate Dr. F. J. Waldo, the City Coroner, on his election to the position of President of the Coroners' Society. Hospital men need no reminding of the importance to them of inquests being conducted by Coroners with hospital experience, though the possession of the double qualification of medical and legal training is gradually becoming general. This was not always so, but to-day it is realised how essential is such knowledge and training for a post which is assuming by degrees the prestige properly attaching to the public services for which it is responsible. Coroners can be of the greatest service or disservice to hospitals according as they understand the hospitals' practice and difficulties, while at the same time in the public interest maintaining a valuable publicity on such questions as deaths under anæsthetics and the like. Dr. Waldo, in his constant endeavour to press successfully the claims to fees of members of hospital staffs when giving evidence in Coroners' Courts, in his evidence before Royal Commissions, in his enforcement of the need for properly appointed courts, *post-mortem* rooms and mortuaries, has done good public service. The new Coroner's Court provided by the Borough of Southwark, in which, as we noted last week, he lately presided for the first time, is at once a tribute to his work and to the recognition of the importance of his office; and it is fitting that the new President of the Coroners' Society should be sitting in a court which is an example for his colleagues to point to throughout the country. It is a graceful fact that the opening of this model court and his election to the office of President of the Coroners' Society should so nearly coincide in point of time.

### THE SPECIAL MEDICAL REPORTS OF THE L.C.C. EDUCATION DEPARTMENT

The amount of defective vision revealed by the report of the public health work of the London County Council, the broad aspects of which were discussed last week, is somewhat startling. Among boys of twelve years of age only 51 per cent. had perfectly normal visual acuity, and among girls of the same age only 43.8 per cent. At the same age in 22.7 per cent. of the boys and 25.7 per cent. of the girls the vision was $\frac{6}{12}$ or worse in either or both eyes. Again, another special investigation of interest is that by Dr. Leipoldt, who examined 3,198 children in order to determine the presence and significance of a floating tenth rib. Professor Stiller pointed out in 1898 that the presence of this abnormality is associated with visceral displacements and general malnutrition. Dr. Leipoldt found Stiller's sign present in 17.6 per cent. of the children he examined; 21.3 of them could be classed as normal, the remainder presented various physical defects, especially signs of malnutrition and pre-tuberculosis. He considers that the prognosis as regards improvement is worse in these cases than in those where this sign is not present. We cannot deal with all the special reports, but the above provide samples of their interest.

### POOR-LAW INFIRMARIES AND MEDICAL SUPPLIES.

The majority of Poor-Law authorities whose contracts for medical supplies terminated on March 31 have now made their arrangements for future supplies. We stated in our issue of January 23 last that it would be advisable and perhaps necessary to have contracts for a less period than twelve months for such things as drugs, surgical dressings, etc., which are liable to fluctuation in prices. This course has been adopted by most Boards of Guardians, though varying methods of procedure have been used by the different Poor-Law authorities. Edmonton Guardians, for instance, have placed contracts for drugs, surgical dressings, etc., for a period of three months, whilst St. Pancras have accepted a contract for a three months' supply of drugs, the surgical dressings and instrument contract being for twelve months. In other cases—as at Wandsworth—the Guardians have asked the old contractors whether they are agreeable to continue the contract on the old terms, any extra charges being adjusted by mutual agreement at the end of the period agreed upon. It seems probable that higher prices will rule for many chemicals hitherto of "enemy country" origin. Whether an adequate supply will be maintained of such articles is a matter of uncertainty, for although sodium salicylate, for instance, is now being manufactured in this country, the quantity available is comparatively small. At the moment, the greatest difficulty is being experienced in obtaining surgical dressings, due both to increased demands for special hospitals and perhaps more to transport difficulties. Where the precaution is taken of labelling consignments for Poor-Law infirmaries "Medical Supplies, Urgent" greater facilities are often afforded for their early delivery.

### THE LONDON MENTAL DEFICIENCY COMMITTEE.

Mr. Cyril Cobb has been re-elected chairman of the Mental Hospitals and Mental Deficiency Committee of the London County Council. Since he became a member of the Council some years ago

Mr. Cobb has always taken a keen interest in the mental hospital work, and during his last term of office as chairman of the Committee he was responsible for the launching of the Council's mental deficiency work. A great deal still remains to be done before the work can be expected to run with perfect smoothness, and it is only fitting that Mr. Cobb should have been reappointed to his onerous office. Mr. H. V. Rowe, last year's vice-chairman, has also been reappointed.

### POOR-LAW FORMALITIES.

At an inquest recently held in Bethnal Green on a child who died from burns, the mother complained that she had taken the child to a hospital and was referred to the infirmary. On reaching the latter she was sent to the relieving officer to obtain an order, and after doing so she was sent back to him because he had omitted to sign the order. She had to carry the child about during all these journeys. The infirmary medical officer said the child's burns had been dressed at the hospital, and the case was therefore not one that could be looked upon as an urgent case demanding immediate admission. The Coroner, however, very rightly pointed out that the mother should have been allowed to leave the child at the infirmary whilst she obtained the order. The objections to such a course are that the relieving officer may take the view that, since the case has been admitted by the medical officer as a matter of urgency, an order cannot be given, and that in some cases the friends, having obtained the admission of the patient, will not take the trouble to try to obtain an order. Many Boards of Guardians also make it as difficult as they possibly can for the medical officer to exercise the power conferred upon him by the Local Government Board to admit urgent cases upon his own authority. What constitutes urgency is, however, a medical matter, and no medical officer should hesitate to run counter to his Board if the fulfilment of the usual formalities entails any unnecessary suffering to the applicant for admission. Should any dispute arise, a frank statement of the principles governing his action disarms criticism.

### CARDIFF MENTAL HOSPITAL AND THE WAR OFFICE.

At the request of the War Office, acting in conjunction with the Board of Control, the Cardiff Mental Hospital Committee have agreed to hand over the institution for treatment of wounded soldiers. The following is a summary of the terms of the transfer. All expenditure incurred or loss sustained by the committee arising out of the use of the hospital by the War Office will be met by the Army Council; the War Office will be responsible for the care and treatment of the soldiers and management of the hospital, which will be handed over as a going concern with the whole of the staff; the War Office will appoint any additional staff required, apart from any extra staff on existing lines necessitated by the removal of patients; scales not less favourable than existing scales and emoluments will continue to apply to the present staff and to future appointments; the medical superintendent will be the officer in charge, under the direct control of the War Office; the whole of the present staff will remain in the employment of the visiting committee, and present powers of dismissal will remain operative; the medical superintendent will make contracts for supply, and otherwise carry on the business side of the administration, will open a separate account, and will present monthly accounts to the War Office through the Board of Control; the farm will continue to be managed by the committee, and the accounts kept separate from those relating to soldiers; produce supplied to the hospital will be debited to the War Office account at reasonable rates; expenditure in respect of the patients retained at the hospital will be charged to the War Office account. The agreement covers the cost of removing patients to other mental hospitals and the charges made for their maintenance.

### THE SCHOOL MEDICAL SERVICE IN BIRMINGHAM.

The temporary school medical officer has prepared for the education committee a report on the medical inspection and treatment of Birmingham school children, which contains several points of interest. In addition to the annual inspection a scheme was devised whereby the assistant school medical officers paid revisits at suitable intervals, so as to see how far the advice given to the parents had been acted upon. Moreover, revisits were also paid by school nurses, and an increasing number of industrial care committees, acting on information supplied to them by the medical staff, were able to visit dilatory parents and urge on them the need and desirability of medical treatment. The moral of the report, based on the facts which are tabulated in it, is that the school medical service supplies the sole efficient means of giving suitable treatment to many of the children. The minor ailments by its means can be subjected to treatment without delay, more serious cases be dealt with in their incipient stage, and thus loss of educational efficiency through absence from school is obviated. The revised scheme of medical inspection and treatment of children leaving school which came into operation in 1914 is described as working smoothly. Owing to distress caused by the war, free breakfasts were provided on Saturdays and Sundays from the beginning of September. The number of these, however, has been since somewhat reduced through the reception of separation allowances by soldiers' wives. The growth and co-ordination of the work are alike remarkable, and the success already attended by it points to the time when a complete medical service will be organised throughout the country by a comprehensive scheme of child-welfare medical agencies up to the age at which the Insurance Act's provisions begin to apply, on the lines, perhaps, of the arrangements already devised at Bradford.

### THE AMBULANCE SERVICE FOR LONDON.

Considerable dissatisfaction has been evinced by many Metropolitan Boards of Guardians at the selection of their institutions by the London County

Council as places to which persons injured in street accidents may be taken. It was suggested in Southwark that the Newington Workhouse and St. George's Workhouse in the Borough should receive these cases, but the Guardians drew attention to the fact that at neither institution had they a resident medical officer nor a nursing staff that could deal with such cases. The result has been that both institutions have been removed from the list prepared by the London County Council of places where those suffering from street accidents may be taken. It is suggested in Lambeth that the infirmary in Brook Street should receive patients, but here, again, the Board of Guardians have raised the objection that their institution is not a hospital, and that people injured in the streets should be taken to the hospitals, and not to the Poor-Law infirmaries. The Board, in a letter to the London County Council, point out that there is a constantly increasing call upon Poor-Law infirmaries to deal with cases of sickness which have hitherto been attended in general hospitals, and they do not regard the infirmary as a proper place for the reception of accident cases unless they are of such serious urgency as apparently to preclude the possibility of the patient being taken to a hospital without serious risk of his life. It was within the power of the medical officer of the infirmary, in the absence of an order from the relieving officer, to refuse to admit a case of accident, unless it be of extreme urgency. The Board consider that it should be the recognised practice of the Ambulance Service in street accident cases to take the patient to a hospital, utilising the infirmaries only in cases where immediate treatment is apparently necessary, and these institutions are the nearest available for the purpose.

### A WHOLE-TIME MEDICAL SERVICE IN BERMONDSEY.

An interesting occasion in the annals of the Poor-Law service took place recently when the late district medical officers, who have relinquished their posts under the new scheme of whole-time medical service to the poor of the parish, were bidden a formal farewell. The Clerk to the Guardians, Mr. E. Pitts Fenton, gave an interesting review of the change. Owing to the appointments of district medical officer, public vaccinator, and medical officer to the workhouse having fallen vacant almost simultaneously in 1913, the Guardians were advised to link up the appointments of district medical officers to the medical staff of the infirmary, and to proceed with this policy as and when vacancies occurred, so that the whole medical service of the parish would be co-ordinated under one responsible head. Eventually the scheme was widened so as to include the other existing appointments of district medical officers. The three remaining district medical officers were therefore brought into the scheme and abolished. Terms were arranged between the parties, which have been since approved by the Local Government Board. Thus it was felt that three officers of long standing should not be allowed to sever their official connection with the staff without some mark of esteem being given to them, and a presentation accordingly took place. The new scheme of a whole-time medical service was admitted by the retiring medical officers to be an administrative advance, since it placed the head of the infirmary in medical control of the district and other institutions of the parish.

### THE VALUE OF HEALTH VISITORS.

The medical officer of health for Manchester, Dr. Niven, has prepared a scheme for the approval of the City Council, which by the establishment of baby clinics would supplement the provision already made in the city for home visitation of infants in the central section of Manchester, and for the supervision of midwives, the treatment of pregnancy, and the consultations with a woman practitioner at the mothers' school. It is proposed to establish four centres where advice and treatment would be given in respect of the ailments of infants and children up to school age. The centres proposed would be elaborate and consist of two adjoining six-roomed cottages adapted for the purpose. On two days a week, in the first instance, medical attendance would be given by a children's physician and a whole-time medical officer at a salary of £350 a year respectively. A nurse would be attached to each centre to attend the clinics, keep records, dispense medicines under medical supervision, and investigate home conditions. The circumstances of patients would be examined and a scale fixed, while those who could afford to pay would be referred to a medical man. The centres, as regards their work, would be correlated with the school for mothers, to avoid overlapping. Dr. Niven expresses agreement with the opinion of the medical officer to the Local Government Board that the most important part of such work is done by health visitors, and the appointment of two additional visitors is recommended. One of the medical officers at St. Mary's Hospital would attend once a fortnight at each of the four centres to advise pregnant women. The present amount spent on child welfare in Manchester is £2,812; the new scheme will cost an additional £4,000, towards which the Local Government Board will probably contribute one-half. The health visitor is once again officially recognised as one of the most useful of public servants, and the new scheme is the best tribute to the value of her work.

### "THE WAY OF THE RED CROSS."

This book, the profits from the sale of which are given to the *Times* Fund for the Sick and Wounded, is serving the double purpose of raising money for a good cause and of bringing home to the public some idea of what the Red Cross is actually accomplishing for the wounded. The publishers, Hodder and Stoughton, and the authors, Mr. E. Charles Vivian and Mr. J. E. Hodder Williams, have done their work very well, and the result is a capital collection of Red Cross incidents, which throw into relief the organisation whose work they illustrate. It is a good half-crown's worth and well combines an account of the organisation involved with the point of view of those who administer and those who benefit by it. As an instance of the former we may note with interest the plea of a member of the administrative staff at the Boulogne base, who, rejected as a recruit, gave up his post as a chartered accountant to undertake the necessary but laborious clerical work. "I think," he is quoted as remarking, "we do our bit down here at the base, and if they give medals to the people in the hospitals we, who are on the administrative staff, ought to have them too." Hospital officers over here will sympathise with that aspiration, and the main value of the book to them and to the public is the way in which the different sides of the Red Cross work are made to live in its pages.

### MENTAL HOSPITALS FOR THE WOUNDED.

Epsom again is figuring in the arrangements for accommodating wounded, though happily on this occasion the question of moving soldiers from quarters already allowed them does not arise. In fact, Horton Mental Hospital, which accommodates 2,000 mental patients, has been taken over by the military authorities. The matter is of special interest from the fact that the London County Council mental hospitals are already sufficiently overcrowded with patients to have led the Council to begin building a fifth mental hospital at Epsom, in spite of local protests from Lord Rosebery and others, who appeared to think that a population of some 6,000 in the existing institutions was sufficient. The patients at Horton, therefore, are being accommodated in two ways. Some of them are being moved to temporary quarters in connection with the Council's eight other mental institutions, while others are being boarded out in the hospitals of other local authorities. Apart from the problems of the displaced patients, modern mental hospitals have much to recommend them as institutions for the wounded. Their big, airy wards, light and cheerful rooms, fine grounds, and recreation halls provide ample space for numbers, and require less adaptation than other types of building not designed for the treatment of ordinary medical and surgical cases.

### DUTY-FREE RECTIFIED SPIRIT IN HOSPITALS.

The question asked by Mr. Bridgeman with reference to the supply of duty-free rectified spirit to hospitals has received the following reply from the Chancellor of the Exchequer: "The general question of the use of spirit is under consideration, but I am afraid that it would not be possible to grant this particular exemption." Mr. Bridgeman: "Is the right hon. gentleman aware that the London Hospital alone has spent £256 on tinctures of this kind, of which £229 is duty, and could he not, at any rate, make an exemption in favour of tinctures and solutions of iodine? I think it will be easier to do that." Mr. Lloyd George: "I could supply the hon. gentleman with the reasons given to me by the Customs as to the difficulty of making a discrimination. I will hand the paper over to him."

### REFUSE AND THE HEALTH OF LONDON.

Vigorous efforts are being made by the public health authorities of London to induce the Government to provide better facilities for the removal of dust and refuse by the railway. In Bermondsey and Islington the situation has become acute as there are vast quantities of material ready for removal, but the services for their transference have been requisitioned by the Government, leaving the refuse in thickly populated parts of the Metropolis to the danger of public health. Borough after borough had joined in the appeal, as nearly all are affected. Some are without dust destructors, and are entirely dependent on the railway or the river for the removal of their material. In some boroughs special printed appeals have been made to the residents to burn as much refuse as they can, especially vegetable matter, and the response has had a splendid effect on the quantity removed. But with a promise of warmer weather the nuisance is likely to become accentuated unless speedy steps are taken to inaugurate a better service of transit. In the interests of public health it is to be hoped that some measures will be devised to remedy the dislocation at present existing.

### EASTER AND THE DRUG MARKET.

Notwithstanding the nearness of the Easter holidays business is in full swing; in ordinary years the week preceding Easter is usually a slack period, but this year is an exception, the reason being that manufacturing druggists and dealers find it no easy matter to keep pace with the demand for drugs for the Medical Services of our own and our Allies' Armies. Acetyl-salicylic acid is again dearer, and most of the synthetic drugs still have an upward tendency in price. There has been no further advance in the value of cod-liver oil; it is impossible to say definitely what will be the future course of the market for this drug. The opinion held by some is that the price will continue to advance as a result of the large demand from Germany; but, on the other hand, it must not be forgotten that the yield of oil from the Norwegian fishings has been good and that at normal times the value would probably have been only about half the figure now quoted. Cocaine has again advanced in price, and it seems probable that we shall see still higher prices. The value of opium is not quite so firmly maintained, but no price reduction of any importance can be expected for some time; indeed, it is not improbable that prices will advance again. Santonin still has a lower tendency in price.

### TO OUR READERS.

Contributions are specially invited from any of our readers to these columns. They should deal with topical subjects and news. They must be authenticated for the information of the Editor only. The minimum payment if published is 5s. There is no hard-and-fast rule as to space, but notes of about twenty lines in length are preferred.

